# Novel morphometric analysis of higher order structure of human radial peri-papillary capillaries: relevance to retinal perfusion efficiency and age

**DOI:** 10.1038/s41598-019-49443-z

**Published:** 2019-09-17

**Authors:** Marconi Barbosa, Ted Maddess, Samyoul Ahn, Tailoi Chan-Ling

**Affiliations:** 1 The Australian National University, John Curtin School of Medical Research, Eccles Institute of Neuroscience, Canberra, 2601 Australia; 2 University of Sydney, Department of Anatomy, Bosch Institute, Sydney, 2006 Australia

**Keywords:** Predictive medicine, Translational research

## Abstract

We apply novel analyses to images of superficial capillaries that are located near and around the optic disc of the human retina: the radial peri-papillary capillaries (RPCs). Due to their unique perfusion of the nerve fibre layer the RPCs are particularly significant for optic-neuropathies. The inputs to the analysis were z-stacks from 3D confocal fluorescence microscopy from 62 human retinas aged 9 to 84 years. Our aim was to find morphometric correlates of age. The retinas had no ophthalmic history. The analysis was undertaken in two stages: (1) converting the z-stacks to 3D tubular networks of vessels, and (2) characterizing the tubular networks using features derived from the Minkowski functionals (MFs). The MFs measure: the capillary volume, surface area, mean breadth, and Euler number. The mean breadth is related to tortuosity, wall shear stress and resistance to flow, and the Euler number is related to the density of loops (collaterals). Features derived from the surface area, mean breadth and Euler number were most related to age (all p ≤ 0.006). The results indicate the importance of pressure-equalizing loops and tortuosity as quantitative measures related to perfusion efficiency. The novel morphometric analysis could quantify disease-related accelerated aging and vessel malformation.

## Introduction

The human retina exhibits an extraordinary level of structural organization while performing a critical metabolic balancing act that is assisted by the retinal vasculature. During embryonic development retinal vessels spread over the superficial retinal surface and then penetrate down through its layers to form an outer (deep) retinal vascular plexus to meet the demanding metabolic requirements of the retina^1–3^. To optimise visual acuity, the retinal vessels should also allow visible light to pass with minimum scatter to arrive at the photosensitive cells^4,5^. Capillary networks present particular issues for the control of perfusion^6–11^ with astrocytes, endothelial cells, pericytes and smooth muscle cells contributing to this regulation.

Traditional modelling of capillary beds focused on bifurcation of vessels^12^, but recently loops have been demonstrated in real capillary networks^13,14^, and we confirm that for the human radial peri-papillary capillaries^15,16^ of this study. Topologically similar shunts or *collaterals* have been reported in heart vessel networks of older subjects^17^. Part of their role might be to reduce *fluid hammer*, as occurs in poorly designed water pipe networks when flows modulate abruptly. Fluid hammer has been reported for cerebral blood vessels^18,19^. Aside from protecting vessel walls from haemorrhage and aneurysms, loops and shunts may also be relevant to the process of angiogenesis during tissue neovascularization^20^. Overall, the question arises as to how to quantify the 3D structure of vascular beds in a way that might encompass important functional variables such as resistance to flow, tortuosity, and the frequency of collaterals. Measures related to the diffusion of metabolites into and out of vascular beds are also of interest. As we show in this study, measures derived from the Minkowski functionals^21–23^ may assist us to quantify metabolically important factors in health and disease.

The intimate relationship between geometry and physiology is evident from the simplest of fundamental concepts such as pressure, which is defined in terms of the *area* over which force is distributed. These relationships occur either directly as a consequence of fluid mechanical forces acting on endothelial cell surfaces with additional influence from cells and basal lamina found on the vessel wall, or are mediated by complex mechanisms, e.g. branching angle, diffusion^8^, atherosclerosis^24^, coagulation^25^, sprouting^26^, branching^27,28^, pruning^29^, tissue regeneration^20^, phagocytosis^30,31^, photobiomodulation^23,32^, aneurysms and stroke^33^. Although these hemodynamic and physiological effects are widespread, there is hardly another organ in which the interplay between structure and physiology is more critical than in the mammalian retina^9,11^. Oxygen is a critical metabolite that is limited by arterial and capillary supply. Diseases with vascular involvement such as age-related macular degeneration, diabetic retinopathy, retinopathy of prematurity, central retinal vein occlusion, and glaucoma are responsible for the majority of untreatable cases of retinal blindness^6,34,35^. Age is the primary risk factor for several diseases. Some diseases, like glaucoma, represent accelerated aging, so the ability to detect normal aging might infer power to detect some diseases. Optic nerve head blood flow is known to decline with age although the cause is uncertain^36,37^.

Here we explore measures related to flow within a retinal vascular plexus, the radial peri-papillary capillaries (RPCs). Their restriction to the retinal nerve fibre layer makes the RPCs uniquely responsible for meeting the metabolic needs of the retinal ganglion cell axons, making their functional capacity particularly relevant to glaucoma^38^ and optic neuropathies^39^. The RPC density is reduced in glaucoma^40,41^. In the present study we employ mathematical generalizations of the notion of area in two dimensions (2D), and volume in three dimensions (3D): namely the Minkowski functionals (MFs) to quantify higher-order RPC structure. We have previously shown the utility of the MFs in obtaining the optimum binarisation of 2D blood vessel images^32^, and their relevance to human discrimination of complex textures^22^. In 3D, there are four MFs: the total volume, the surface area, the mean breadth and a topological quantity, the Euler number. The mean breadth is the surface integral of the mean curvature, which captures aspects of vessel tortuosity^42^, and is also indicative of wall shear stress, which is directly related to resistance to flow^43,44^. The 3D Euler number is the number of connected components minus the number of tunnels plus the number of cavities, which in the context of vascular beds characterizes the distribution of loops in the vascular network. Importantly such loops can act to regulate pressure and provide even diffusion of metabolites^45,46^. The close link between the MFs and the physical properties of materials is well documented in the physics and material science literature^21,47–52^. The MFs have been applied less often to images of 3D structures in biology and medicine, a notable exception being bone morphometry and its relation to mechanical fatigue^53,54^.

In this study, we present a framework for quantitative morphological analysis of vessel arbors with the aim of relating aspects of morphology with function. We explored the effects of age using confocal z-stack images of fluorescently labelled radial peripapillary capillaries in presumptively normal human retinas. We examined samples from 62 human retinas that ranged in age 9 to 84 years. The novel analysis introduced here has two basic steps. First, the stacks of confocal images from retinal wholemounts are segmented to accurate 3D representations of the tubular vascular plexus. Then the 3D data is quantified in terms of the generalized volumes, i.e. the 3D Minkowski functionals. Measures derived from the MFs (features) were then examined for their relationships with age.

## Methods

### Preparation of human retinal wholemounts and staining of radial peri-papillary capillaries

Eyes from a 9 year old child and 61 adult eyes (aged 17 to 84) with no history of prior ocular disease were collected in accordance with the Declaration of Helsinki and approved by the University of Sydney Human Ethics Research Committee (Protocol 15190) and examined using immunohistochemistry. All human specimens utilised in this study were obtained by donation to Professor Tailoi Chan-Ling by the NSW Tissue Banks | Australian Ocular Biobank | NSW Organ and Tissue Donation Service. All specimens were donated to the NSW Organ and Tissue Donation Service with written informed consent from a parent and/or legal guardian as appropriate and donors over the age of 18 provided written informed consent for the use of their own tissue. The corneas were removed for corneal transplantation with the same written informed consent as above, following which the posterior ocular segments are donated to Professor Chan-Ling for research purposes. All specimens when received from the Eye Bank come with a medical record sheet. This lists: cause of death/co-morbidities, time of enucleation and ophthalmic history. All specimens with any recorded history of ophthalmic conditions including diabetic retinopathy, wet age-related macular degeneration (wet AMD), dry AMD or geographic atrophy, hypertensive retinopathy and glaucoma were excluded from the study. Those retinas are directed to other studies with those requirements. Patients with a recorded systemic record of Type-1 or Type-2 diabetes or hypertension were similarly excluded from the study. Unfortunately the donor service records did not consistently report record things like smoking history.

The postmortem delay prior to fixation varied between 18–32 hours. The posterior eyecup are immersion fixed within 15 minutes of removal of the corneas for transplantation. The eye is kept at 4 degrees Celsius at all times and tissue samples were analysed within 5 weeks of donation where possible.

The protocol was optimised by conducting a dilution trial (from 1:50 to 1:10,000) on small pieces of peripheral retinal beyond the area occupied by the RPCs. Once the protocol was optimised as detailed above, all specimens in the study were processed in the same way irrespective of cause of death or age of the specimen. It was evident that the background fluorescence was higher in the aged specimens and the tissue was typically more fragile with age. Utilising the offset available on the confocal microscope, to improve the signal to background ratio on specimens with higher background fluorescence, making it possible to analyse all specimens included in this study.

**Figure 1:**
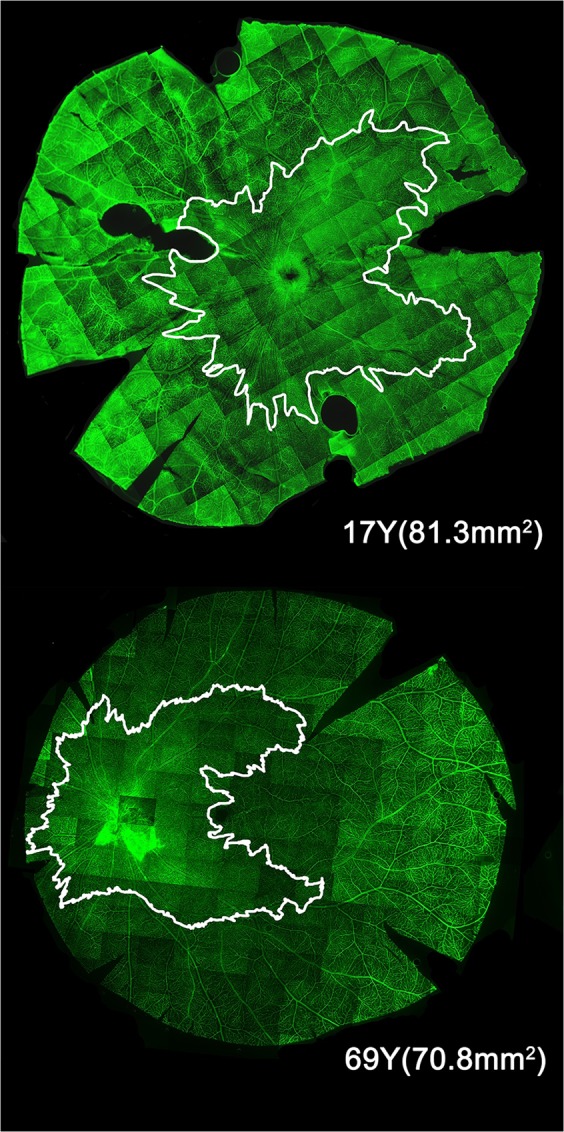
The radial peri-papillary capillaries (RPCs) of the human retina are located around and near the optic disk (Papilla). For these whole-mount retinas the white border indicates the maximum extent of the RPCs. The outer boundary of the RPCs is indicated by the irregular white line. The two examples indicate that there is some heterogeneity of the RPC domains.

Retinal wholemounts (Fig. 1) were dissected as previously reported^55^, and stained with *Ulex Europeaus* Agglutinin-1 (UEA) Lectin^56^. Radial incisions were placed through the entire thickness of the retina, choroid and sclera. The choroid and scleral attachments were dissected away to ensure continuity of the retinal wholemount through the optic nerve head. A 5 mm skin biopsy punch (Proscitech, Kirwan, QLD AU) or a custom built 20 mm stainless steel trephine were used to punch out either a 5 mm circle centered over the optic nerve head or a 20 mm circle centered over the full extent of the RPC distribution from the nasal side of the optic nerve head to the fovea.

Tissue was incubated for 48 hours at −4 °C with the UEA Lectin FITC conjugated (L006, 1:100 diluted in 0.05% Triton X-100 in PBS (Sigma Aldrich Corp., St Louis, MO, USA), washed with 0.1% Triton X-100 in PBS. Retinal wholemounts were mounted with the ganglion cell layer up in Prolong Anti-Fade (Invitrogen, Mount Waverley, Australia). Each retinal wholemounted area of the retina was captured utilizing a Zeiss PALM Fluorescent microscope with 5× objective. The entire retinal area within the 20 mm Diameter punched area was captured utilizing the mosaic function of the Zeiss confocal software requiring over 200 scans to visualize the entire vascular tree over this area. Mosaic tiling, stitching, shading correction was done with the Zeiss post-capture analysis software. We marked the outer limits of individual RPCs, and joined the marks to form an outer perimeter of RPC distribution (Fig. 1).

The RPCs are unique in their structure and rheology in that they comprise a vascular plexus that is restricted to the nerve fibre layer of the retina with only capillary sized vascular segments, where there are no arterial or venule calibre vessels in this plexus. For this reason, the fields of view selected were representative of the vascular bed analysed and they represent all the RPC vessels. For the reason that this vascular plexus only has capillaries making it easier to analyse. This structure is readily observable in our previous studies^57–59^.

### Z-Scans of the human radial peri-papillary capillaries for further automated morphometric analysis

Fluorchrome-UEA stained retinal wholemounts were viewed using a Zeiss META LSM500 confocal inverted microscope (Carl Zeiss, Germany) with a 20× objective, equipped with appropriate excitation lasers. Alexa 488/FITC fluorescence was excited at 495 nm. Two variables were found to vary between the analysed specimens. First, background tissue autofluorescence differs significantly with pathology and aging; second, the human RPC distribution in the z-plane varies between individuals and with topography. For these reasons, the following protocol was developed and adhered to for all specimens to minimise these influences during our analysis. The observer scanned the retinal wholemounts over the entire region where RPCs are located and determined the upper and lower limits of the confocal microscope setting to ensure all x-y planes containing RPCs are contained within the volume of tissue scanned (z-axis). The average number of z sections was 24 sections encompassing the thickness of the RPC layer (23.7 µm).

Four fields of view (FOVs) were selected per specimen and were collected in two regions: the superior temporal and inferior temporal lobes, which represents 1–2 o’clock and 4–5 o’clock for the left eye for example. Two FOVs were imaged in each region 3–5 mm away from the rim of the optic nerve head. Each FOV was captured using the Zeiss META LSM500, with real dimensions of 450 µm × 450 µm × 1 µm per focus plane, which translated to 1024 × 1024 pixels per focus plane (each voxel being 2.276 × 2.276 × 1 pixels/µm). Z-stacking was used to capture individual planes of focus in series. The tile scan function in the Zeiss LSM software was used to montage adjacent maximum–intensity projections of z-stacks. Z-stacks from 4 adjacent regions were stitched to form one confluent area for further analysis as detailed below.

### Preliminary analysis: transformation to accurate 3D representations of the tubular vascular plexus

**Figure 2:**
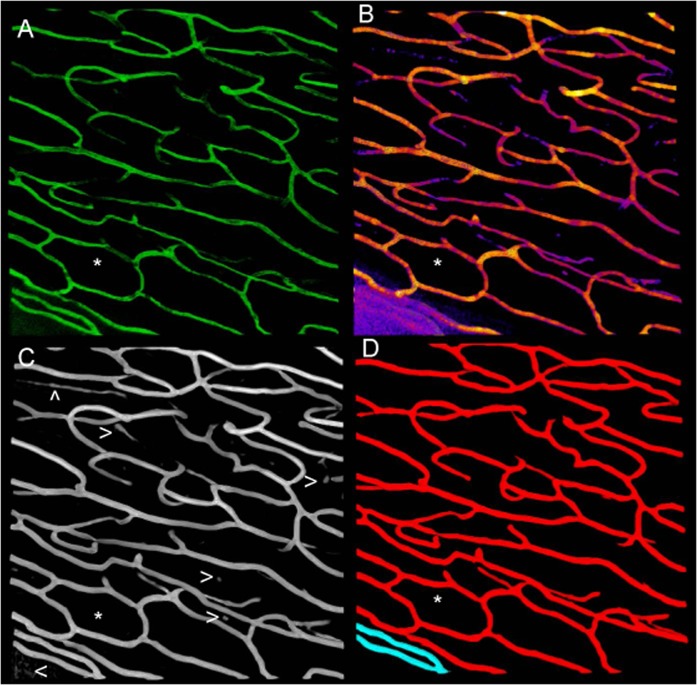
Processing examples for a sample from an 18 year old male. (**A**) shows the projection of an original confocal Z stack using the Fiji 3D-Viewer method^60^. (**B**) Local Thickness as illustrated with a colourmap (hotter colours are thicker vessels). (**C**) The output of the Adaptive Structure Filter of the same stack. (**D**) The two large connected elements of C (red and cyan). The outlier elimination step has removed some of the smaller noisy elements seen in C (arrows). A true loop, confirmed by section of the raw data (A) and the 3D tubular network (D), is marked with a * in A to D. See also Figs 3 and 4.

The z-stack data was initially visualised using the 3D-Viewer capacity of Fiji (Fiji Community: version 1.51 s using Java 1.8.0_66 for Linux). This tool operates in two basic ways^60^. The first is to set the transparency of pixels according to their intensity, black pixels being completely transparent. This allows our visual systems to interpret what is essentially a cloud of points, as a semitransparent 3D object. It is important to understand that the result is not a quantitative 3D description or model of the object. Figure 2A shows an example 3D-Viewer projection from our data. In a second step the 3D-Viewer can create a dense network of triangles, the faces of which can be rendered to particular colours and transparencies. The process of generating the net is very general and does not include any knowledge of the type of object being imaged. These are excellent general tools for visualising 3D data of unknown form. Our objective here is to use the knowledge that the capillaries are tubular and exploit that information to transform the z-stack data into accurate tubular models, and to then do a novel morphometric analysis of the resulting 3D tubular vessel networks. Having done that we used 3D-Viewer to visualise our models, and we eliminated a few data sets where staining, focus or other histological sampling errors were too great. Almost 1 FOV in every 4 retinas was discarded but every retina had at least ¾ of its data.

Following initial visualisation we used a Fiji plugin to perform what is called a Local Thickness Analysis^61^. This analysis seeks to represent the cloud of z-stack data as the concatenation of a dense set of spherical shells, each sphere representing a local diameter. Shells smaller than a certain size, which are enclosed within larger ones, are discarded. This very general method can be used to provide the local distance between surfaces (thickness) of quite arbitrary shapes: bubbles, parallel lamella, tubes, etc. Although potentially useful, the method does not use knowledge about particular types of 3D structure, such as the branching tubes encountered in capillary networks, which could permit more accurate representations to be obtained from the intrinsically noisy data. An example of output from the Local Thickness method is illustrated with a colourmap in Fig. 2B (hotter colours represent relatively thicker vessels).

### Minkowski functionals

The fundamental principles behind the Minkowski functionals (MFs) were introduced and applied to pattern characterization by various researchers^21,47–49,52,62–64^. Central to this approach is the reliance on measures obeying the principle of *additivity* for two samples A and B: V(A ∪ B) = V(A) + V(B) − V(A ∩ B). It is intuitive that the area and volume obey these additive relations because they are based upon counts of the pixels or voxels of interest. Like the area and volume all the MFs obey the additivity principle. The MFs have been called by a variety of names^21,47–49,52,62–64^ including: additive functionals, intrinsic or generalized volumes, and quermassintegrals. In two dimensions (2D) the three Minkowski functional (MFs) are: the area, perimeter and a quantity called the *Euler number*. In 3 dimensions the four MFs are the volume, the surface area, a quantity referred to as the *mean breadth*, and the 3D Euler number. For 2 and 3 dimensional data the MFs represent counts of progressively higher order statistical/structural relationships between neighboring pixels/voxels in the object of study. The higher order MFs are thus higher-order mathematical generalizations of area and volume. Being topological measures, the units of length of the sides of the pixels/voxels are largely meaningless. Collectively the MFs provide a comprehensive description of aggregative, spatial stochastic processes that modulate complex system geometry^65–67^. The mean breadth has no 2D counterpart. It is the integral of the mean 3D curvature, which is conceptually related to tortuosity, and computationally to resistance to flow within a tubular network. The Euler number is a measure that: in 2D counts the number of connected components minus the number of holes; and in 3D gives the number of connected components minus the number of tunnels and plus the number of enclosed cavities. In the present context the Euler number is related to collaterals within the capillary plexus. The mean breadth and Euler number play key roles in this study.

It is worth mentioning that other measures of structural complexity, such as the fractal dimension^68^ and *lacunarity* (which we have publish on^66^) are non-additive. Thus, lacunarities measured for two samples cannot be combined to give a meaningful average lacunarity. The Euler number captures similar information but the additivity principle means that the mean Euler number for two or more samples (or other linear combinations of Euler numbers) is well defined, just as mean volumes are.

### Adaptive structure filter – finding tubes

An accurate segmentation and object identification is vital in any morphometric analysis and we dedicate a significant level of attention to it here. The main procedure here is based upon the relative ratio of the Hessian matrix similar to Frangi’s vesselness^69^, which adaptively boosts the appearance of tube-like structures. As a starting point, we applied a single structural filter based on the relative magnitude of the Eigen-values of the Hessian operator applied to the convolution of the original image with a 3D Gaussian. The variance of this Gaussian distribution was chosen to match the mean calibre of the vasculature from the Local Thickness analysis. This single filter has a limited ability to resolve variations in calibre. An improved *adaptive structure filter* was generated by a point-wise logical operation in which at each voxel the largest signal is selected from the outputs of multiple structural filters tuned to different calibres about the initial estimate. An example of the output is shown in Fig. 2C. It is worth noting that while the Fiji-based thickness analysis has difficulty with the noisy capillaries at bottom left of Fig. 2B (purple in colour), the adaptive structure filter identifies the tubular vessel well (Fig. 2C). Following that step, a standard connected component procedure is followed by outlier elimination – both in voxel values and in the size of the clusters. Overall tubular structure is emphasized for the full range of vessel sizes and non-tubular structure is removed. Figure 2D, shows the connected tubular elements of Fig. 2C, the very small isolated elements (white arrowheads in Fig. 2C) having been removed at this final step. In short, this sequence of image processing segments the vasculature, *utilising the prior knowledge that the regions of interest are tubular*. The results are large, mainly connected, tubular networks. This segmented structure is now unambiguously represented by connected voxels with 1 representing vessels, and 0 representing everything else. Details of the adaptive structural filter methods are given elsewhere^70^.

**Figure 3:**
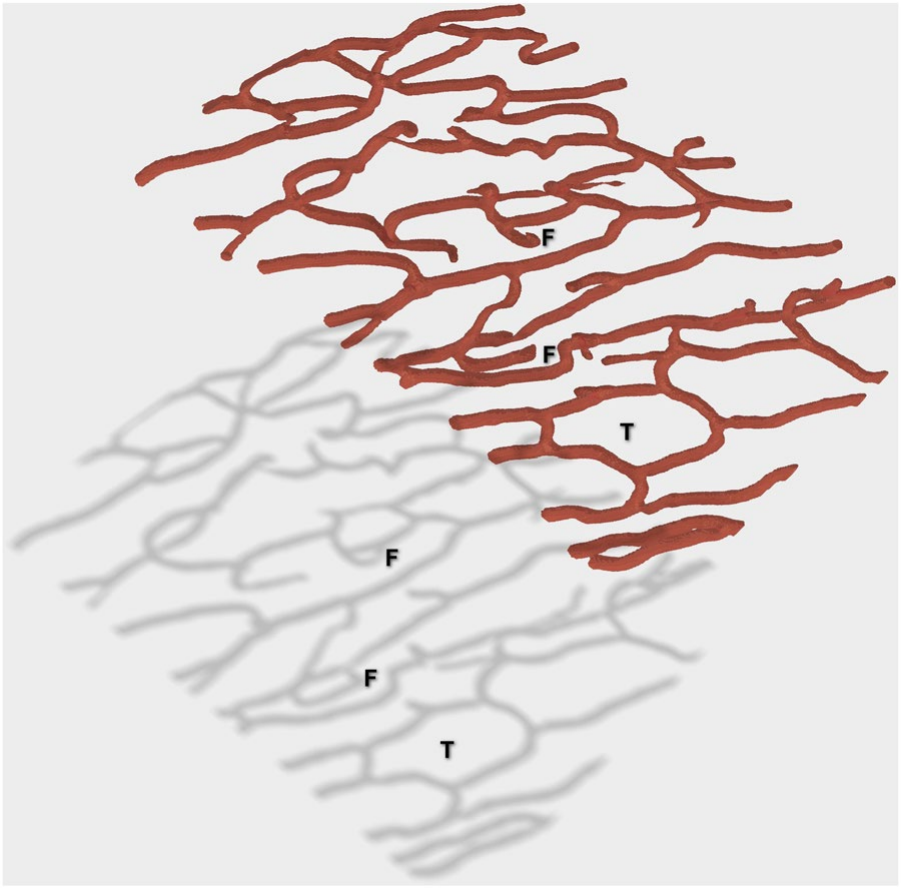
Vessel projections and true and false loops. The red tubular network represents capillaries recovered from the Adaptive Structure Filter method taken from Fig. 2D. Below it is a grey surface reflection. The reflection illustrates that 2D-projections of 3D vessel networks can be misleading, with several false putative loops appearing (F). By contrast the red tubular network model allows us to conclude that only one true loop is present (T).

Some advantages of having a true 3D representation of capillaries are shown in Fig. 3. The figure shows a sub-region of a capillary network as obtained by our methods direct from confocal Z-stack imaging of the human adult RPCs. Below it, is a surface reflection. The reflection represents a possible interpretation of the network if viewed as a 2D projection. The shadow (projection) suggests several false loops (F), while the 3D network reveals only one true loop (T). It is also clear that if the local thickness analysis step either over- or under-estimated the capillary diameters, this could create false features or make features disappear. The initial thickness analysis produces a robust estimate of the best size and so, as a consequence, such problems are unlikely. However to control for any such errors we introduce a quantitative dilation and inflation of the capillary networks over a wide range of scales, and calculate the Minkowski functionals at each scale. We refer to this as *Dilation Analysis*. Among other things this allows small vessels to contribute if their structure is important.

### Dilation analysis

**Figure 4:**
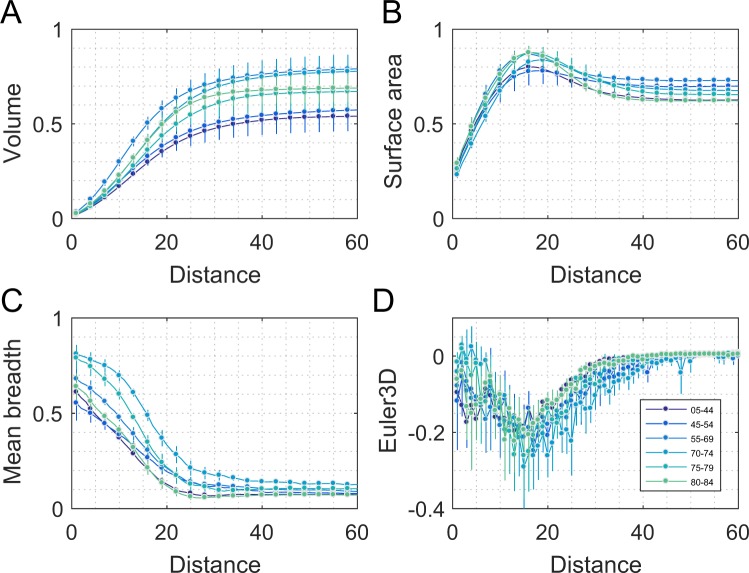
The dilation signatures of the four 3D Minkowski functionals for different age groups. Dilation of vessel diameter proceeds via an Exact Euclidean Distance transformation. Dilation distance (vessel radius) is given on the abscissas is in voxels. Note that 0 indicates the original segmented data and larger numbers are inflations of the data. The four panels illustrate the dilation signatures of the four Minkowski Functionals: (**A**) the Volume, (**B**) the Surface Area, (**C**) the Mean Breadth, and (**D**) the Euler Number. The curves are the averages (±SD) for the standard six age groups (legend). The age groups contain a median of 10 retinas each (Methods). The same groups are used in other figures to facilitate comparisons. Age appears to modulate several of the dilation signatures of the Minkowski functionals. Similar curves were created for each retina and then 10 features (parameters) are extracted from each curve (Table 1). The data of panels A to C were normalized to the maximum of all the data, and for panel D the scaling is to the total range of the data. In A to C the mean ± SD for every third point are shown to allow the smooth curves to be seen. In D the mean ± SD for all dilations is shown.

With a clear binary representation of the vasculature, we analyse the geometrical and topological aspects of a sample of the vessel network based on the Minkowski functionals. In our framework, we are interested in the scaling behaviour of these morphological descriptors, so that our analysis is robust with scaling in the same spirit as the box-counting procedure for estimating the fractal dimension^68^. Hence, we calculate all four 3D MFs, for each sample, across a series of dilated copies at a wide range of possible vessel diameters. The dilation is done via an Exact Euclidean Distance Transform. If we image the capillaries as tubular balloons, the dilation is akin to inflation of the balloons. This procedure yields four morphological *signatures* as a function of the dilation scale (vessel radius), one for each MF (Fig. 4). Further details of these methods are given elsewhere^70^. The study of the scaling behaviour of the MFs amounts to a significant extension to the fractal analysis^71^. Next, we quantified the above signatures by a set of 10 *features* such as moments and critical points of the signature functions (e.g. the mean, variance, slopes, maxima and minima). The signature functions quantified were like those in Fig. 4 but generated for each retinal sample. Table 1 defines the 10 features measured per MF, and gives the shorter labels used in subsequent descriptions. We initially examined the 40 features using principal component analysis (PCA) in order to estimate the proportion of variance explained by a limited subset of the features, and the relative importance of particular MF features of each of the principal components to describing age of the samples. The 40 features were also examined in terms of their utility for describing age using ANOVA testing and a cross-validated form of stepwise regression.

**Table 1:** Features of the Minkowski Functionals computed across the range of dilations of the signature functions (e.g. Fig. 4).

	NAME	Variable Computed Across the Range of Dilations
1	var	Variance
2	std	Standard Deviation
3	mean	Mean
4	median	Median
5	max	Maximum
6	maxScale	The abscissa value of the Maximum
7	min	Minimum
8	minScale	The abscissa value of the Minimum
9	dmax	Maximum of the derivative of the curve
10	dmaxScale	The abscissa value of dmax

In Fig. 4 the data are shown binned into the six age groups (5–44, 45–54, 55–69, 70–74, 75–79, 80–84 years of age). These ranges were chosen to make the group statistically comparable. Thus the six age groups contain a median of 10 retinas each (range 9 to 12) and the age group means are shown with ± SD. The same age groups and colour scheme are used in other figures to facilitate comparisons.

The Exact Euclidean Distance transform is not only a mathematically simple way to add multi-scale support for the morphological analysis, the set of transforms also represents the oxygen profile in the extracellular medium because it approximates a linear diffusion process: a linear function of the shortest distance to the object at each point in the extra-cellular space. The resulting extra-vascular space is generally a disconnected set of regions, which are represented by logical false (0) in the vessel image, but are not empty spaces. These *void spaces*, i.e. the distribution of cavities, tunnels etc., are important physiologically as they constitute the very reason the blood vasculature arrived there in the first place – these spaces accommodate cells to which oxygen and nutrients are to be delivered. The Minkowski Functionals quantify these spaces at the same time.

The computations for the Adaptive Structure Filters, Minkowski Functionals and the Dilation Analyses were undertaken in Matlab version 2016b (The Mathworks, Natick MA). The Principal Curve analysis used the Princurve package of R version 2.15.0 (Free Software Foundation, Boston MA).

## Results

**Figure 5:**
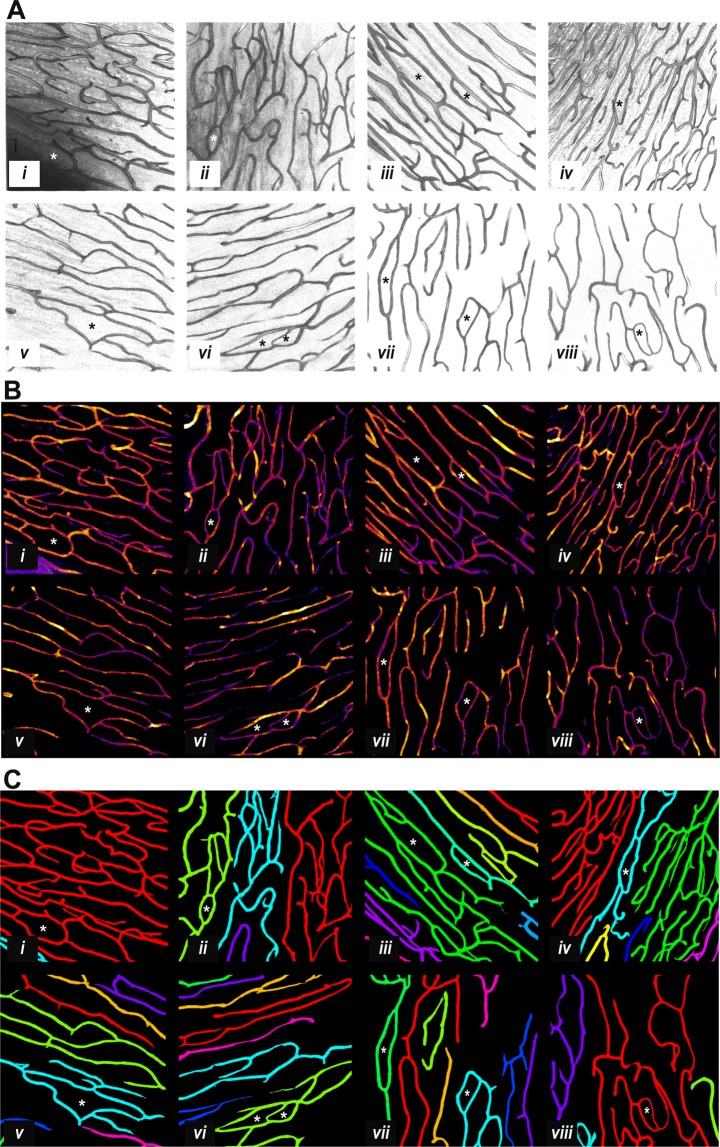
Examples of radial peri-papillary capillaries by age. Panels A to C represent the processing steps of Fig. 2A,B,D for eight retinas (*i* to *viii*). Donor ages from *i* to *viii* were respectively: 18, 31, 34, 55, 63, 63, 71, 72 years. In each of A to C retina *i* is the same as for Fig. 2. The 34 year old (*iii*) was female, the rest were males. (**A**) The Z-stack data were visualized with the Fiji 3D-View method^60^ and presented here as 2D projections. The intensity map has been inverted compared to Fig. 2A in order to highlight the (now dark) speckle noise in the raw images. Examples of true loops are marked with *. (**B**) The thickness data using the same colour map as Fig. 2B. (**C**) The final segmented capillaries produced by the Adaptive Structure Filter method as in Fig. 2D.

Figure 5 shows examples of the appearance of the RPCs as a function of age presented for the main processing steps illustrated by Fig. 2. For each of panels 5A to 5C the retinas *i* to *viii* were taken from persons of age: 18, 31, 34, 55, 63, 63, 71, and 72 years respectively. There are some possible regions of capillary drop-out in the samples from retinas *vii* and *viii* but otherwise it is not clear that the images represent different ages. Examples of true loops, confirmed by inspection of the 3D data (e.g. Fig. 3), are indicated by *.

**Figure 6:**
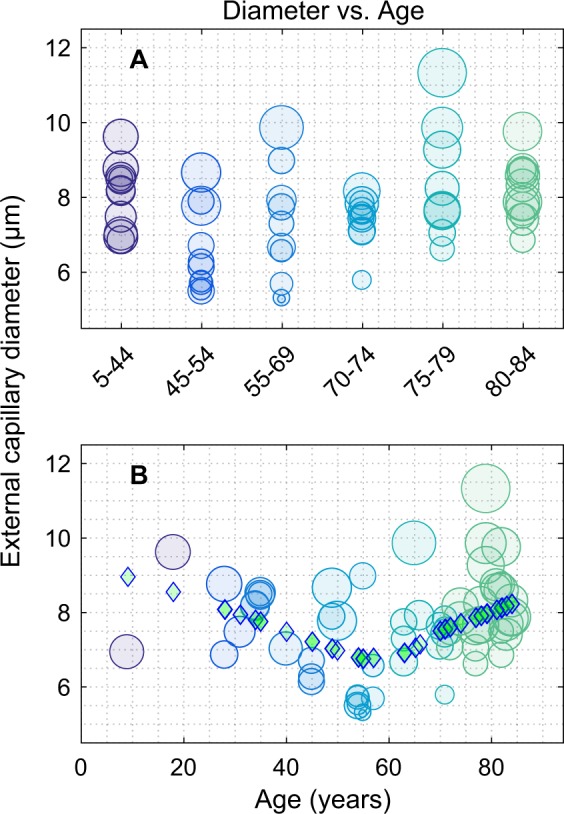
Local Thickness for each eye as a function of age. The ordinate is the mean vessel diameter for a given retina (stack), and the standard deviation for each stack is coded by the circle diameter. (**A**) The data are shown binned into the six age groups of Fig. 4 (median 10 retinas/group, range 9 to 12). The distribution of diameters appears to be poorly related to age. (**B**) Here the diameters are shown on a linear age scale. The diamonds are the result of a Principal Curve analysis, which suggests a concave trend with age having an inflection point around 60 years. In A and B the dot colour codes age group membership in the same way, and is the same coding as for Figs 4, 8 and 9.

Figure 6 summarises the results of the Local Thickness analysis (Methods, Figs 2B, 5B), showing that vessel diameter (calibre) does not appear to have a linear relationship with subject age, matching another recent report^72^. Each dot in Fig. 6 is based on a median of 1.1 million measured diameters/retina, and the dot diameter is proportional to the standard deviation. In Fig. 6A the ages are binned into the same six age groups as in Fig. 4 with a median of 10 of retinas per group. Figure 6B shows the same data on a linear age-scale. The diamonds are the result of a Principal Curve analysis^73^, which attempts to find the spine of the data cloud for which there are an equal number of points in directions orthogonal to the spine. The output produced one diamond for each of the dots (eyes). The curve of diamonds suggests an initial downward trend with increasing age, followed by a rise in diameter after an inflection point at about age 60. Attempts to fit separate linear relationships above and below the inflection point did not yield significant slopes. Thus, the principal curve analysis should be taken as the best estimate of a trend. Support for the trend comes from other data presented below that display more robust convex or concave relationships with age.

**Figure 7:**
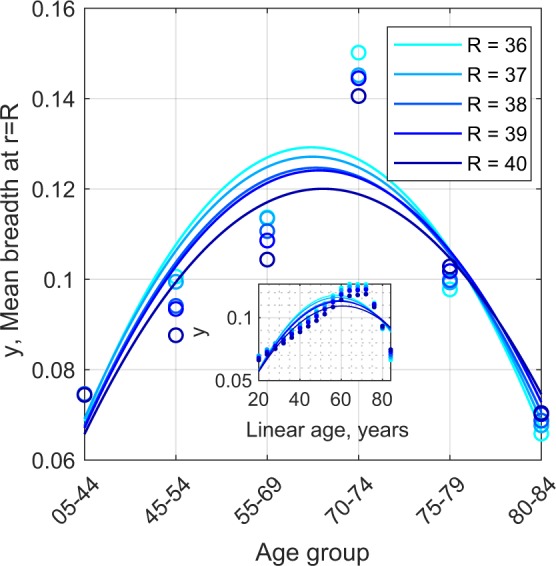
The values of Mean Breadth at specific dilation points, or distances from the central vascular skeleton. The value of R in the legend relates to the range of dilations (radius in voxels as on the abscissa of Fig. 4) using the exact signed Euclidean Distance transform. The ordering of age groups is consistent across the range of dilations, all showing a maximum mean breadth around 70 years. The continuous curves are polynomial fits to the data (symbols). The inset shows the fitted curves on a linear axis.

Figure 7 illustrates a sampling of the mean breadth data of (Fig. 4C) as a function of age group. The peak is at about 70 years. The figure also illustrates that mean breadth varies quite smoothly as a function of age group, and therefore the conclusion that the peak is near 70 years is quite robust. This smooth and robust aspect arises from the Dilation Analysis (e.g. Fig. 4) which makes the analysis tolerant of the error in the segmentation process.

**Figure 8:**
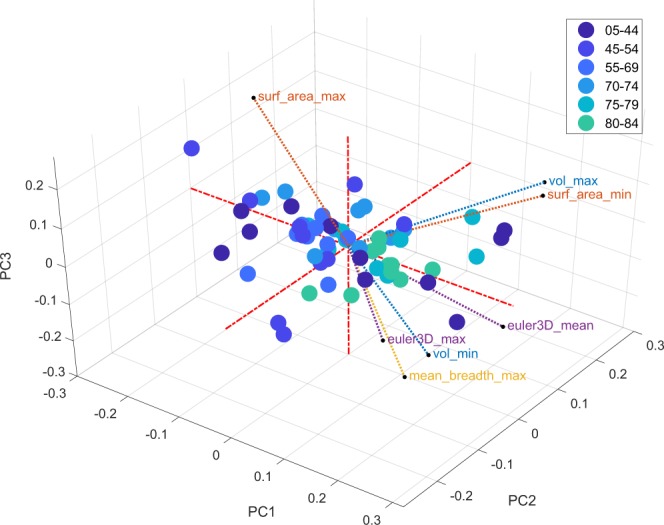
The first three principal components of all considered Minkowski morphometric features. The results show a significant explanation of the total variance in the data by the first three PCA components. The colours of the dots (representing each retina) are the same as Figs 4 and 6, and indicate that the PCA 1 axis corresponds well to age. The first PCA component also display strong correlation with several features of the Mean Breadth and the 3D Euler Number, as illustrated by the labelled vectors with the smaller black-rimmed white dots at their end indicate the direction and magnitude of those and some other original feature variables. The symbols illustrate that measures like the maximum value of the 3D Euler number (cf. Fig. 9) is well aligned with component 1.

We next calculated a set of features that quantified dilation data like that of Fig. 4, but which were derived for each retina. These ten summary measures included the standard deviations, maxima, minima, etc. (Table 1) for each of the Minkowski functionals evaluated across the range of evaluated dilations. The resulting 40 features (per retina) were transformed to z-scores and were then entered into a principal components analysis (PCA). Principal components are weighted sums of all the data, which indicate the major axes of variation of that data. Almost all the data (67%) is captured by just the first two principal components. The third component only accounts for another 5% of the feature data. Plotting the retinas in the space of the first three components (Fig. 8) shows that component 1 is well associated with age (dot colour). Some of the MF features are shown on the same axes (labelled vectors ending in circles). Component 1 is closely aligned with several higher-order features like the maximum and mean of the Euler number, and also the maximum of the mean breadth. This indicates that these features on their own are well associated with age.

**Figure 9:**
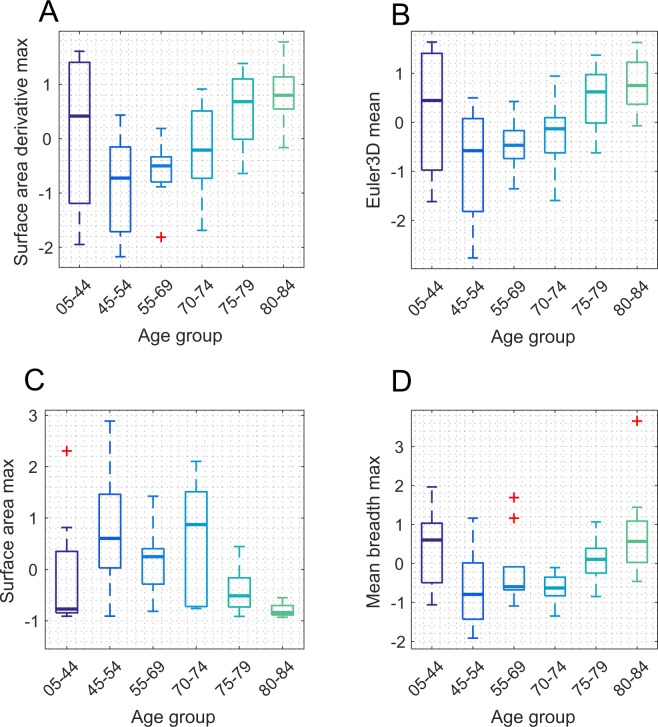
Four morphometric features associated with age from the PCA analysis. The four best measures show a convex (**A,B,D**) or concave (**C**) relationship with age with an inflection point around 55 to 60 years (cf. Fig. 6B for vessel diameter). The maximum value of the derivatives of the dilation signature for Surface area (**A**) seems to show the strongest, and most linear, relationship with age beyond about age 50. The mean of the Euler number is similar (**B**). The age groups used here are the same as in Figs 4, 6A and 7. These provide a median of 10 eyes/boxplot so that the estimates of the percentiles of the boxes plots are uniformly well statistically based. Note that the three oldest age groups are in linear steps of 5 years.

The behaviour with age of four of most promising features from the PCA is shown in Fig. 9. Their curvilinear relationships with age are reminiscent of the mean breadth itself (Fig. 7), and to some extent the capillary diameter (Fig. 6B). The maximum value of the derivatives of the dilation signature for surface area (surface area dmax, Fig. 9A) seems to show the strongest, and most linear, relationship with age beyond about age 50. The mean of the Euler number has a similar form (Fig. 9B). Recall that the Euler number relates to the number of loops, and that the mean breadth relates resistance to flow. *Thus, the number of loops and the resistance to flow appear to rise in later year*s.

As a further exploratory analysis we examined the significance of those four features by making multiple ANOVA comparisons (with Bonferroni correction) between age groups defined in 5-yearly cohorts. All four provided significant discrimination of the 80–84 year group from one or more of the 45–54, 55–69 or 70–74 groups (Tables S1 to S4).

From about age 40 onward many of the Minkowski features showed a near linear age-dependence. We therefore investigated these 52 eyes using a bootstrap/cross-validated form of stepwise regression fitting them to age. All 40 Minkowski features were entered into the analysis. The bootstrap method used 3000 cycles of random sampling with replacement of the feature data for the subjects, with the samples entered into stepwise regression on each cycle. That process was then repeated 6 times, each with a different set of 3000 random samplings of the feature data for the 52 eyes. Each of the 18,000 stepwise regressions selected particular features (p for inclusion of 0.05, exclusion 0.1). The 25^th^, 50^th^, and 75^th^ percentiles of the number of features selected per regression were 2, 3, and 4 features. These 2 to 4 feature models had r^2^-values of 0.626 ± 0.089 (mean ± SE), and median F-statistics of 28.7 (p < 10^−9^). The frequency at which each feature appeared was quantified for those 2/3 of the fits. The 10 most frequently selected features were highly stable, the first 5 being selected at the same frequency order across the 6 repeats (Table 2). Occasional exchanges of order among the next six features were corrected to match the most common order. The selected features overlapped considerably with those of Fig. 9, even though the age-group considered was somewhat different to that for the PCA. In particular surface area dmax (Fig. 9A) was selected in 81.4% ± 0.46% (mean ± SD) of the conservative 2 to 4 feature models. The second-most common volume-based measure (8.9% of fits) was the variance in the volume signature function, i.e. a second order measure of volume. The simpler means or medians of the volumes occurred in <0.5% of fits. Overall several higher order measures of the peri-papillary capillary structure were well correlated with age, and linear functions of volume were not.

**Table 2:** Top 10 frequencies of the Minkowski Functional features (out of 40) selected by the bootstrapped stepwise regression.

Minkowski Feature	% Frequency ± SD	P
surf_area_dmax	81.6 ± 1.71	<0.00001
surf_area_minScale	49.5 ± 1.47	0.009
euler3D_std	22.9 ± 1.35	0.003
euler3D_mean	11.0 ± 0.74	0.006
euler3D_minScale	10.9 ± 0.52	0.007
euler3D_dmaxScale	9.3 ± 0.38	0.011
vol_maxScale	9.3 ± 0.61	<0.00001
vol_var	8.9 ± 0.63	0.006
euler3D_min	6.8 ± 0.54	0.010
mean_breadth_var	6.5 ± 0.28	0.002

The low variability in the selection rates being indicated by the frequency SDs. Data are for the 2/3 of conservative models that selected 2, 3 or 4 feature variables. These models had r^2^-values of 0.626 ± 0.089 (mean ± SE). The p-values shown are the medians for that variable across linear models in which they were selected.

## Discussion

The importance of mechanical stability in a dynamic fluid network cannot be overstated. As early as 1808, Thomas Young pointed out the effects of sudden change in fluid pressure on elastic tubes^74^. This effect would later be known as *fluid hammer*. Blood is composite and non-Newtonian, but subdued fluid hammer is still present in blood vessels^18,75^. It may be important in age-related atherosclerosis, which stiffens vessel walls making them more vulnerable to the hammer effects in case of flow obstruction^18,33,76^. Loops or collaterals in vessel networks may provide a simple mechanism for pressure equalization and redundancy in case of sudden blocks. Loops may also help balance oxygen tension across the network. Striking oxygen tension gradients are a feature of mammalian retinas^6,10^ and come about by the interplay between oxygen supply, intravascular and tissue parenchyma, and consumption by the resident cells.

Loops are present in all levels of the human and rodent retinal vascular beds and are important in angiogenesis^3,20,77^. As mentioned, even fairly recent models of capillary beds do not include loops^12^, but recently loops have been demonstrated in real human^3^ and rat^20^ retinal capillaries, chick chorioallantoic membrane capillaries^13,14^, and coronary collaterals within the human heart^17^. We certainly saw them in our samples of the human retinal RPCs (asterix in Figs 2–4). Here we introduce the 3D Euler number, which quantifies the density of loops, and show that features based upon the Euler number are predictive of age in human retinal peri-papillary capillary beds (Figs 8, 9, Tables 2, S2).

To increase the magnitude of the Euler number of the vasculature, with a fixed number of components (which was assured during our segmentation procedure) either the number of cavities (holes) must decrease, and or the number of tunnels (or loops) increase during the dilation procedure. Models of blood flow through tissues composed of parallel networks of interconnected looping capillaries show that interactions between such capillaries control determine capillary permeability and even-out dispersion of things like blood tracers and metabolites throughout the tissue^45,46^. Thus the correct density of loops is likely to be critical to good tissue health. Formation of novel capillary loops has been reported for healing of small lesions^20^. Loops might also form in response to other insults like lowered oxygen tension, perhaps leading to an increase in loop density with age.

Features related to the mean breadth explained a reasonable part of the variance in the dataset, being predominant in the first principal component (Fig. 8). These rose with age from about 40 years onwards (Fig. 9D). Recall that the mean breadth is related to vessel tortuosity^42^, and in being a measure of wall shear stress is a direct measure of resistance to flow^43,44^.

There was also a trend towards after an initial fall in diameter at younger ages followed by increasing vessel diameter in the over 60 group (Fig. 6B). A possible cellular basis for this finding comes from our earlier studies showing that pericyte/endothelial ratio in rat retinal vessels decrease with age^59^ and that the ratio of astrocytes to neuron also decrease with age in rat retina^78^. Since astrocytes mediate neurovascular signalling to capillary pericytes^79^ it is possible that the decrease in mean thickness up to 60 years of age is due to pericyte loss and compromised pericyte/astrocyte regulation of blood flow with age, since both cell types decline with age. Given some evidence for nonlinear relationships between capillary data and age (Fig. 6B) we examined generalizations of linear models to explore the relationship between age and the Minkowski signature features. We employed various non-linear link functions and non-normal underlying distributions for the data, and explored those models with interactions of the features up to fully quadratic models. The change of goodness of fit did not justify the complexity of the models.

A feature of the RPCs is that while they are too small to be resolved in fluorescein angiography, they can be studied using OCT angiography (OCTA)^80^ in living persons. OCTA is a rapidly developing field^81^. Presently the most common OCTA output is the *retinal capillary density*^38,80^ which is proportional to retinal nerve fibre layer thickness in glaucoma^38^ and optic neuropathies^39^. That is purely an area-based measure, the lowest-order Minkowski functional for 2D data, and so does not capture any higher order structure. Our preliminary data indicate that OCTA is likely to benefit from analysis of the 2D and 3D MFs. In particular those data show that higher order MFs measured from capillary data derived for histological and OCTA from the same patch of retina provide similar results, both providing highly correlated information on capillary structure as a function of retinal depth^70^.

Although care was taken not to include donors suffering from eye diseases (Methods), it is inevitable that older people will have potentially undiagnosed conditions. Diabetes was excluded. Another potentially asymptomatic disease of concern is hypertension (HT). Prevalence rises rapidly in persons over 60 and ¾ of our subjects were 78 or over (18 eyes) about 10 to 12% of whom would be expected to have uncontrolled HT in Australia. That being said none of our retinas had HT retinopathy so we suspect it would be less than 5% so, ≤1 eye. The Eye Bank records did not give smoking history, which is a concern, however in Australia the prevalence of smoking in the 65 to 74 cohort is 9% and for over 75 s is 4.3%.

To circumvent these limitations future studies should include a much larger dataset, even if it means lower resolution images from living people possibly provided by OCTA^70^. We could then search for relationships between morphological alterations associated with various diseases. Animal models could be useful in this search but there are some species differences. We have reported on aspects of rat retinal pericyte and smooth muscle biology^9,59^ and they do not have RPCs. Also their superficial capillaries exhibit some contractile properties^82^.

Some possible limitations of our statistical methods should be described. In respect of Fig. 6B the Principal Curve analysis indicates a trend towards capillary diameters that fell until age 55 or so and then rose. That is an exploratory method however and so the result is open to interpretation. We did attempt to fit various models with different slopes on either side of a range of inflection points but none yielded significant slopes. For the bootstrap/cross-validated stepwise regression method of Table 2 we reported on conservative models that selected between 2 and 4 variables in their step-wise linear models. Across the 6 independent cycles of cross-validation these models represented the 25th to the 75th percentiles of the number of fitted variables. We also tried somewhat broader range of models and this did not markedly change the types of frequency of the variables selected.

The segmentation method is an improvement on that of Frangi^69^. A limitation of that method is that is set to recover a single scale of vessel diameter. Here we use similar calculations but at a range of scales of tube size and segmentation was then based upon the best scale in terms of the strongest signal at each voxel. The mathematical details are given elsewhere^70^. The best value was selected as the maximum of log of the estimates at each scale. While this was more robust a possible limitation is how well does the method function for smaller vessels as the vessel scale approaches the sampling density? We have addressed this elsewhere for two cases were the tube size was smaller relative to sampling. One case was histological data of 46 retinal ganglion cells of 4 types. Applying the same method segmented the cells well and MF based features allowed excellent classification of the 4 types. In that study we also examined retinal vessels sampled from the same retinas using both high resolution histological data (as here) and coarsely sampled and noisy OCTa data. The method recovered the vessels well from both data sets and the MF features were very similar. This might suggest that at least for high resolution data that the method is robust. We did however eliminate a small number of outliers, both in terms of both in voxel values and in the minimum size of the clusters of voxels. These steps eliminated tiny, isolated, non-tubular features so are unlikely to have affected small vessels.

Overall it was interesting that some features of higher-order Minkowski functionals of the RPCs were modulated by age. Disease can sometimes represent accelerated aging. For example the peri-papillary retinal nerve fibre layer thins with age^83^, but not as fast as in glaucoma^84^. This might suggest a role for the RPCs in glaucoma. Blood flood decreases with age in the central retinal artery^36,37^ and optic nerve head tissue^36^, and RPC density drops in advanced glacuoma^40,41^. Changes to loop density and surface area and may also be relevant to the process of angiogenesis during neovascularization^20^, as seen in diabetic retinopathy and age-related macular degeneration. Therefore one would expect that measures based upon the higher-order MFs may characterise these more dramatic vascular changes. Age appears to strongly modulate higher-order MFs of retinal peri-papillary capillaries.
